# Nudging toward vaccination: a systematic review

**DOI:** 10.1136/bmjgh-2021-006237

**Published:** 2021-09-30

**Authors:** Mark Donald C Reñosa, Jeniffer Landicho, Jonas Wachinger, Sarah L Dalglish, Kate Bärnighausen, Till Bärnighausen, Shannon A McMahon

**Affiliations:** 1 Heidelberg Institute of Global Health, Ruprecht-Karls-Universität Heidelberg, Heidelberg, Germany; 2 Department of Epidemiology and Biostatistics, Research Institute for Tropical Medicine, Muntinlupa City, Philippines; 3 Institute for Global Health, University College London, London, UK; 4 International Health Department, Johns Hopkins University Bloomberg School of Public Health, Baltimore, Maryland, USA; 5 School of Public Health, University of the Witwatersrand, Johannesburg-Braamfontein, South Africa; 6 Harvard Center for Population and Development Studies, Harvard University, Cambridge, Massachusetts, USA

**Keywords:** public health, health policy, systematic review, vaccines

## Abstract

**Background:**

Vaccine hesitancy (VH) and the global decline of vaccine coverage are a major global health threat, and novel approaches for increasing vaccine confidence and uptake are urgently needed. ‘Nudging’, defined as altering the environmental context in which a decision is made or a certain behaviour is enacted, has shown promising results in several health promotion strategies. We present a comprehensive synthesis of evidence regarding the value and impact of nudges to address VH.

**Methods:**

We conducted a systematic review to determine if nudging can mitigate VH and improve vaccine uptake. Our search strategy used Medical Subject Headings (MeSH) and non-MeSH terms to identify articles related to nudging and vaccination in nine research databases. 15 177 titles were extracted and assessed following Preferred Reporting Items for Systematic Reviews and Meta-Analyses guidelines. The final list of included articles was evaluated using the Mixed Methods Appraisal Tool and the Grading of Recommendations, Assessment, Development and Evaluations framework.

**Findings:**

Identified interventions are presented according to a framework for behaviour change, MINDSPACE. Articles (n=48) from 10 primarily high-income countries were included in the review. Nudging-based interventions identified include using reminders and recall, changing the way information is framed and delivered to an intended audience, changing the messenger delivering information, invoking social norms and emotional affect (eg, through storytelling, dramatic narratives and graphical presentations), and offering incentives or changing defaults. The most promising evidence exists for nudges that offer incentives to parents and healthcare workers, that make information more salient or that use trusted messengers to deliver information. The effectiveness of nudging interventions and the direction of the effect varies substantially by context. Evidence for some approaches is mixed, highlighting a need for further research, including how successful interventions can be adapted across settings.

**Conclusion:**

Nudging-based interventions show potential to increase vaccine confidence and uptake, but further evidence is needed for the development of clear recommendations. The ongoing COVID-19 pandemic increases the urgency of undertaking nudging-focused research.

**PROSPERO registration number:**

CRD42020185817.

**Key questions**

**What is already known?**

- Nudging interventions addressing vaccine hesitancy primarily stem from high-income countries. Little information is available from low- and middle-income countries, many of which are currently facing declining public confidence in vaccines and pointed interest in potential interventions.

**What are the new findings?**

- Nudges that entail changing vaccine defaults (opt-out), giving incentives, and providing reminders and recalls (directly generated from the central data or delivered by trusted health authorities via text messages, emails or personalised letter) have proven efficacious in some settings, are considered viable options when communicating vaccination schedules, and could bolster intention to vaccinate.

**What do the new findings imply?**

- An in-depth understanding of the complexity of the target population is highly recommended to ensure the acceptability and effectiveness of nudging interventions.
- In light of the COVID-19 pandemic and public discourse around risks and chances of novel vaccines, exploring new pathways for interventions, including nudges, that foster vaccine trust and confidence is required. This review provides a comprehensive starting point for such research.

## Introduction

Vaccines have a tremendous impact on disease control and are one of the world’s leading public health achievements, responsible for the eradication of smallpox, the prevention of lifelong disabilities, and major reductions in childhood morbidity and mortality.^1 2^ Vaccination is also a key element for controlling the ongoing COVID-19 pandemic.^3^ However, a growing body of literature highlights that vaccination coverage for an array of vaccine-preventable illnesses, is stagnating or declining globally.^4^ Furthermore, studies that examine routine vaccination for adults (influenza vaccine) and vaccines administered later in life (human papillomavirus (HPV), pneumococcal, and herpes zoster vaccines) also indicate challenges with uptake.^5–7^

Vaccines have become a source of anxiety among many families and individuals globally due to scientifically unfounded fears that they can cause autism and other health problems, and other concerns about vaccine safety.^8 9^ Vaccine hesitancy (VH), defined as ‘the reluctance or refusal to vaccinate despite vaccine availability,’ has gained recognition globally as a top threat to global health, as it risks undermining successful and cost-effective vaccination programmes worldwide.^10^ Contrary to common portrayal, vaccination attitudes are more nuanced than the duality of provaccination and antivaccination attitudes,^11^ and antecedents of VH range from a lack of knowledge and awareness, to culturally rooted misgivings, to concerns regarding vaccines’ short-term or long-term side effects.^4^ VH scholars and stakeholders are increasingly acknowledging this spectrum and noting that successful strategies require an appreciation of the VH continuum, ranging from complete acceptance to active refusal.^12–14^ Mistrust in vaccines, including concerns regarding safety and effectiveness, has spread on several social media platforms (eg, Facebook and Twitter) thereby amplifying the antivaccination movement.^15 16^

The degree of decline in vaccination varies between and within countries, but its potential to overrun health systems due to outbreaks of vaccine-preventable diseases is most acute in low- and middle-income countries (LMICs),^17^ which has led to numerous calls to identify approaches that increase or maintain vaccine coverage at levels that ensure population immunity.^18^

Several health promotion approaches have been proposed and tested to address VH, including video-based vaccine promotion and educational messages in the form of anecdotes, storybooks, and mobile applications.^9 19–22^ A majority of these interventions were focused on changing attitudes and behaviours pertaining to vaccine misinformation and/or safety concerns.^23^ While some of these interventions show promising results in increasing vaccine confidence and uptake, a systematic review conducted in 2013^18^ highlighted that studies were often underpowered and mostly provided observational or indirect evidence for intervention effects. Similarly, a more recent discussion paper^24^ noted that many interventions that aim to change an individual’s perceptions of and feelings toward vaccination demonstrate limited effectiveness.

In contrast to changing attitudes, another stream of health intervention research emphasises ‘nudging’ or changing the environmental context in which a decision is made or behaviour is enacted.^25^ Using nudging, a person’s behaviour can be predictably modified towards a desired end-point.^25^ Examples of nudges include the rearrangement of food on supermarket shelves to place healthy options at eye level (increasing healthy nutrition), displaying arrows leading to sinks in public restrooms (increasing handwashing), or pasting stickers in the centre of men’s urinals (decreasing urinal spillage due to men aiming at the stickers).^26–28^ Evidence suggests that instead of allocating considerable cognitive resources to make a ‘rational’ or ‘smart’ decision, people tend to choose what is easily accessible.^28 29^ In this vein, nudging has been shown to be effective in the context of various health promotion strategies such as increasing healthy food choices and dietary change,^30 31^ self-management of chronic diseases,^32^ and improving HIV and malaria testing.^33 34^

To date, a systematic evaluation of nudging’s efficacy, feasibility, and acceptability in terms of how it affects vaccine knowledge and awareness, intentions and behaviours is lacking. To address this gap, we conducted a systematic review mapping existing evidence regarding nudges’ acceptability, feasibility, and effectiveness in increasing knowledge, intentions, and behaviours (including uptake) related to vaccines. This review aims to contribute to the literature on nudging interventions in this field, to inform policy discussions, and to provide insights around whether and to what degree nudging can affect vaccination decision making.

## Methods

### Design

We employed a systematic literature review methodology^35^ in accordance with the principles of the Preferred Reporting Items for Systematic Reviews and Meta-Analyses.^36^

### Protocol registration

We searched the International Prospective Register of Systematic Reviews (PROSPERO) and Cochrane database for protocols matching this study’s title, aim, and objectives, or for any previously published primary research and reviews on the subject matter. Both databases had no ongoing or published systematic review about nudging in promoting childhood and adolescent vaccine uptake. We registered a protocol for this review on PROSPERO (registration ID: CRD42020185817).

### Search strategies

The two lead authors (MDCR and JL, the latter a biomedical librarian by training) discussed the key words and core concepts. Further, to avoid missing studies that did not directly draw on nudging literature but whose interventions aligned with the selection criteria, we included Medical Subject Headings (MeSH): *‘behavioral control’*, ‘*decision making*’, *‘vaccination’,* and *‘vaccines’*, as well as the following non-MeSH terms: ‘*behavioral intervention’*, *‘behavioral strategy’*, *‘nudge’* and *‘nudging’,* using Boolean operators ‘AND’, ‘OR’ and ‘NOT’ to combine keywords for the final search strings (online supplemental table 1). We excluded the MeSH term *‘immunization’*, defined as the ‘deliberate stimulation of the host’s immune response’,^37^ because our emphasis was on behaviour rather than the biological processes by which a body acquires immunity.

10.1136/bmjgh-2021-006237.supp1 Supplementary data

**Figure 1:**
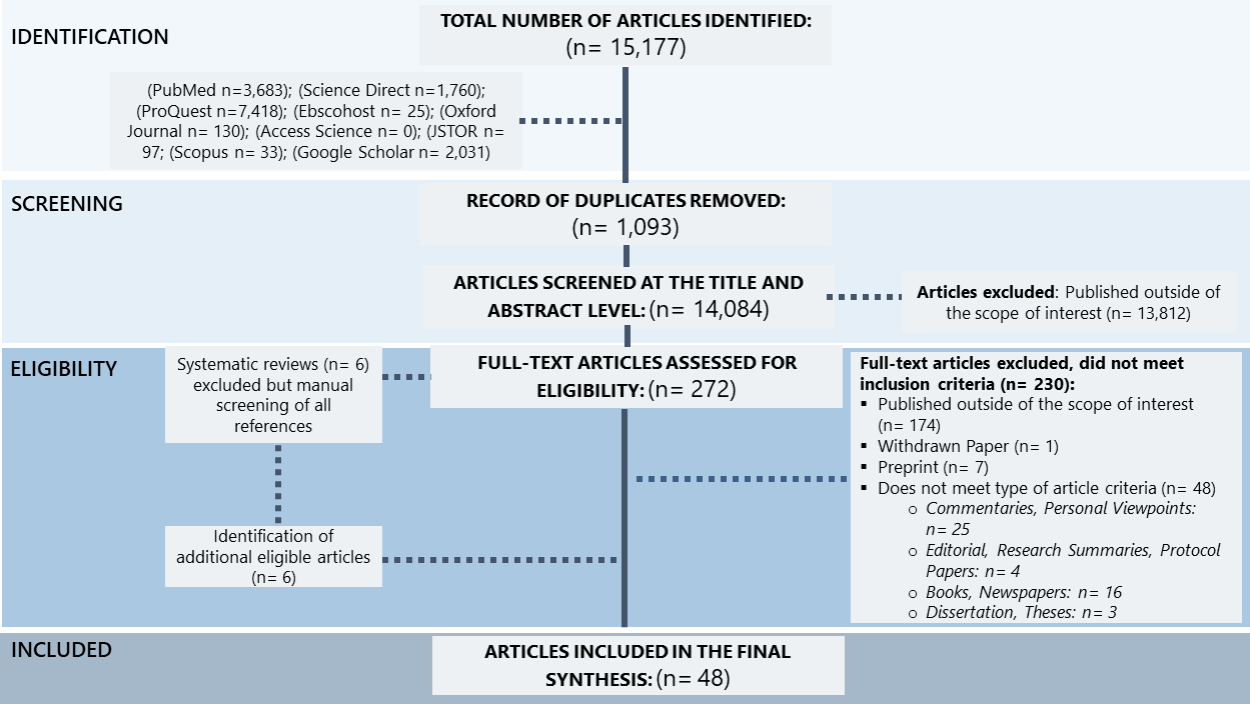
Flowchart of the selection process.

The two lead authors pilot tested the final search strings in two research databases (PubMed and Science Direct) to identify potential problems. We searched nine platforms on both commercial and non-commercial databases to ensure that we captured academic and grey literature (online supplemental table 1). JL independently ran the finalised search strings in the selected research databases (figure 1). MDCR then reran the final search strings in five of the research databases for validation purposes. There were no discrepancies or disagreements noted.

### Study selection and data extraction

MDCR and JL conducted data extraction and validation of included studies. Results from all search strings were initially tabulated per database, including duplicates between strings and between databases. All relevant papers collected were stored in a citation manager (EndNote, V.20) and Microsoft Excel (Microsoft corporation, V.2019).

Titles and abstracts of papers were screened by MDCR and JL based on predefined eligibility criteria (table 1). Initially, we prioritised the inclusion of randomised controlled trials (RCTs), but as the screening identified an inadequate number of RCTs, non-randomised trials (including those in which baseline data were collected before exposure), quantitative descriptive studies, qualitative studies and mixed-methods studies were also considered for inclusion. Full texts of articles were screened in cases where titles and abstracts were not sufficient to clearly indicate exclusion. As part of full-text screening, several articles were found focusing on nudging approaches for adult vaccination. In light of the global urgency regarding the COVID-19 vaccine roll-out, we broadened the scope beyond childhood vaccination. MDCR and JL reran the full-text screening to ensure that all relevant articles were included. This change in review scope was also included in the PROSPERO registration.

**Table 1:** Inclusion and exclusion criteria

Criterion	Inclusion	Exclusion
Population and sample	General population, to reflect all vaccination target groups and all stakeholders involved in the vaccination decision (eg, parents, healthcare workers and adolescents).	
Literature focus	Original research and/or scientific papers on nudging interventions* to address vaccine hesitancy and/or increase vaccine uptake. Studies set within the national immunisation programmes, defined as those vaccines recommended for all age groups—children, adolescents and adults. Evidence regarding the effectiveness of vaccine-related nudges, as well as how the general population experiences and accepts such interventions.	Studies without ethical approval. Articles that are research summaries, commentaries, peer-reviewed conference or seminar papers, and/or personal viewpoints. Articles that are abstract only and/or lack retrievable full text.
Time period	January 2008†–May 2021	
Language	English	

*Nudging interventions as defined by the MINDSPACE framework^41^ and complementary literature,^42 43^ which group interventions according to whether they change messenger, incentives, norms, defaults, salience, priming, affect, commitments or ego.

†Nudging theory emerged in 2008.^25^

We excluded systematic review articles that were captured during the full-text screening, but we manually screened all references listed in these systematic reviews to ensure that no relevant articles were missed during the initial search. In cases of discrepancies, JW served as a third reviewer and articles were discussed until consensus was reached.

### Quality appraisal

The final set of included studies underwent independent quality assessment by MDCR and JL. The assessment was carried out on study level, focusing on study designs and whether study aims and objectives were achieved, adequately reported and synthesised. The Mixed Methods Appraisal Tool (MMAT) V.2018 facilitated the process.^38^ Scores were assigned to each paper per design category and detailed score sheets were saved in Microsoft Excel (online supplemental tables 2–6). Additionally, we used the Grading of Recommendations, Assessment, Development and Evaluations (GRADE) guidelines to assess all certainty of outcomes (ie, consistency, precision, directness and magnitude of the observed effect) of all included RCTs on a high, moderate, low or very low scale.^39 40^

### Collating and summarising

Data collected from the included studies were synthesised and summarised. Relevant data were extracted using a predesigned and piloted evidence summary tool, with categories based on the aims and objectives of the study and additional information on themes emerging during analysis. JL and MDCR piloted the extraction form using five randomly selected studies from all study design groups within the final list of included studies; this process found consistency across the results.

### Data analysis

We prepared descriptive summaries, followed by presentation of results in a tabulated format (eg, including study design, publication details and outcome measures). We followed the MINDSPACE framework^41^ as well as more recent MINDSPACE modifications^42 43^ to structure and categorise the type of nudging approach across the studies.

We performed a mixed integrative and interpretative synthesis of the findings from included studies, structured around the type of nudging employed, ways the intervention was implemented and any measure of impact of the intervention.^35^ We present an in-depth descriptive analysis of the selected studies with an aim to highlight nudges that promote vaccine uptake.

## Results

We performed all searches from 15 May 2020 to 31 May 2021 and identified 15 177 articles (figure 1). After removing 1093 duplicates, the titles and abstracts of 14 084 articles were screened, of which 272 were included for full-text screening, leading to the exclusion of another 230 articles. We identified six additional relevant studies as part of our manual screening of all references listed in six relevant systematic reviews. A total of 48 peer-reviewed articles were included in the final analysis.

### Study characteristics

Key characteristics of the 48 included studies such as study population, sample size and outcome measures are detailed in table 2. A majority of studies (46 of 48) were conducted in high-income or upper middle-income countries (USA (n=31), UK (n=5), China (n=3), Australia (n=2), Japan (n=2), Italy (n=1), Israel (n=1) and Ireland (n=1)). Only 2 studies were conducted in LMICs (one in Bangladesh and one in India). A majority of studies (28 of 48) are RCTs testing interventions, with 4 RCTs piloting an intervention or assessing its feasibility. The remaining 20 articles include 9 non-randomised designs (quasi-experimental, observational and cross-sectional studies), 4 descriptive studies (surveys and quality improvement), 5 mixed-methods design and 2 qualitative studies. A total of 27 studies incorporated control groups that received no intervention or standard of care. A total of 12 studies were published before 2015. All included studies were published as research articles in peer reviewed journals, with more than one study being published in *Vaccine* (n=8), *Pediatrics* (n=3), *PLoS One* (n=3), and *The American Journal of Public Health* (n=2).

**Table 2:** Characteristics of eligible studies

Nudging approach	Authors (year, journal)	Study location	Study population	Sample size	Study design	Target disease	Outcome measures*
Make available information salient	Lwembe *et al* (2016, *BMC Family Practice*)^46^	UK	Parents or caregivers of children under 5, policymakers and practitioners	31 parents,15 policymakers and 9 practitioners	Qualitative design: FGD, telephone interviews	Not specific (vaccine-preventable childhood diseases)	Perception of the intervention (+)
	Borg *et al* (2018, *Vaccine*)^47^	Australia	Parents or guardians of children (6 months–<5 years)	5534	RCT	Influenza	Vaccination uptake (influenza vaccine) (0)
	Uddin *et al* (2016, *Vaccine*)^53^	Bangladesh	Pregnant women, mothers with children aged 0–11 months, and Expanded Program on Immunisation (EPI) service providers in study areas	4158	Non-randomised (quasi-experimental)	Not specific (vaccine-preventable childhood diseases)	Vaccination uptake (all childhood vaccines) (+)
	Ahlers-Schmidt *et al* (2012, *Vaccine*)^54^	USA	Parents of newborns	90	RCT (pilot study)	Not specific (vaccine-preventable childhood diseases)	Vaccination uptake (all childhood vaccines) (−)
	Kim *et al* (2018, *JAMA Network Open*)^56^	USA	Adult patients	96 291	Quantitative descriptive study (Quality improvement)	Influenza	Vaccination uptake (influenza vaccine) (+)
	Patel *et al* (2017, *Journal of General Internal Medicine*)^55^	USA	Adults	45 926	Non-randomised (observational study)	Influenza	Vaccination uptake (influenza vaccine) (+)
	Changolkar *et al* (2020, *PLoS One*)^57^	USA	Primary care physicians from 10 primary care practices	56	Non-randomised study (quasi- experimental)	Influenza	Vaccination uptake (influenza vaccine) (+)
	Duvall (2019, *Pediatric Nursing*)^58^	USA	Families of paediatric inpatients older than 6 months of age, physicians and nurses	173	Quantitative descriptive study (quality improvement)	Influenza	Vaccination uptake (influenza vaccine) and perception of intervention (+)
	Milkman *et al* (2011, *Proceedings of the National Academy of Sciences of the USA (PNAS*))^59^	USA	Employees aged >50 years	3272	RCT	Influenza	Vaccination uptake (influenza vaccine) (+)
	Kempe *et al* (2013, *American Journal of Public Health*)^60^	USA	Parents of children who were aged 19–35 months	31 567	RCT	Not specific (vaccine-preventable childhood diseases)	Childhood immunisation status (up to date) and cost-comparison benefit (+)
	Szilagyi *et al* (2020, *JAMA Internal Medicine*)^61^	USA	Patients in 52 primary care practices	164 205	RCT	Influenza	Vaccination uptake (influenza vaccine) (+)
	Porter *et al* (2018, *Vaccine*)^63^	USA	Parents with daughters aged 9–17 years	761	RCT	HPV	Intention to vaccinate and vaccine confidence (−)
	Saitoh *et al* (2017, *Human Vaccines and Immunotherapeutics*)^62^	Japan	Pregnant women	160	RCT	Influenza and PCV13	Parental attitudes and beliefs towards vaccines and their infant’s vaccination uptake (0)
	Joseph *et al* (2016, *Clinical Pediatrics*)^64^	USA	Mothers with daughters aged 11–15 years	200	RCT (pilot study)	HPV	Vaccination uptake (HPV vaccine) and knowledge about vaccines (−)
	Staras *et al* (2021, *BMC Public Health*)^48^	USA	Parents of adolescents aged 11–12 years	286	RCT (feasibility trial)	HPV	Feasibility and acceptability of vaccine reminders (+)
	Lorini *et al* (2020; *Vaccines*)^50^	Italy	Nursing home workers	1998	Mixed-methods study	Influenza	Intention to vaccinate (influenza vaccine) (+)
	Schmidtke *et al* (2020, *BMJ Quality and Safety*)^52^	UK	Healthcare workers	7540	RCT	Influenza	Vaccination uptake (influenza vaccine) (−)
	Chen *et al* (2020; *Behavioural Public Policy*)^51^	USA	Adults aged 65–70 years	208 867	RCT	Influenza, tetanus, shingles and pneumonia	Vaccination uptake (influenza, shingles, tetanus, pneumonia vaccine) (+)
	Milkman *et al* (2021, *PNAS*)^49^	USA	Patients in 2 large health facilities	47 306	RCT	Influenza	Vaccination uptake (influenza vaccine) (+)
	Maltz and Sarid (2020, *Medical Decision Making*)^66^	Israel	Adults aged 18–65 years	3271	RCT	Influenza	Intention to vaccinate (influenza vaccine) (+)
	Panozzo *et al* (2020, *Journal of Adolescent Health*)^65^	USA	Mothers with children aged 11–14 years	762	RCT	HPV	Intention to vaccinate (HPV vaccine) (−)
Change the way outcomes are framed	Lechuga *et al* (2011, *Annals of Behavioural Medicine*)^77^	USA	Mothers with daughters aged 9–17 years	150	Mixed-methods design (formative and experimental study)	HPV	Intention to vaccinate (HPV vaccine) (+)
	Fahy and Desmond (2010, *Irish Journal of Medical Science*)^76^	Ireland	Mothers with daughters aged 8–16 years	72	Non-randomised study (single group pre-test and post-test)	HPV	Attitudes towards vaccine and intention to vaccinate (HPV vaccine) (0)
	Liu *et al* (2019, *Patient Education and Counselling*)^79^	China	Women aged 18–45 years	453	Quantitative descriptive study (Survey)	HPV	Intention to vaccinate (HPV vaccine) (0)
	Frew *et al* (2014, *Vaccine*)^81^	USA	Pregnant women	272	RCT	Influenza	Intention to vaccinate (influenza vaccine) (−)
	Hendrix *et al* (2014, *Pediatrics*)^82^	USA	Parents with an infant <12 months of age	802	RCT	MMR	Intention to vaccinate (MMR vaccine) (+)
	Motta *et al* (2021, *Frontiers in Political Science*)^44^†	USA	Adults	7064	RCT	COVID-19	Intention to vaccinate (COVID-19 vaccine) (+)
	Freeman *et al* (2021, *The Lancet Public Health*)^78^	UK	Adults (vaccine hesitant)	18 855	RCT	COVID-19	Intention to vaccinate (COVID-19 vaccine) (+)
	Xu *et al* (2020, *Aslib Journal of Information Management*)^80^	China	College students	300	Non-randomised study (quasi-experimental)	HPV	Intention and information need to vaccinate (HPV vaccine) (+)
	Okuno *et al* (2021, *Pediatrics International*)^83^	Japan	Mothers	81	RCT	*Haemophilus influenzae* type b (HiB) and pneumonia (PCV)	Intention to vaccinate (HiB and PCV vaccines) (−)
Invoke social norms	Lee *et al* (2018, *Applied Nursing Research*)^84^	USA	Mothers and daughters aged 14–17 years	18 dyads (mother and daughter)	Mixed-methods design (community-based participatory research with pilot RCT)	HPV	Intention to vaccinate and vaccination uptake (HPV vaccine) (0)
	Attwell and Freeman (2015, *Vaccine*)^85^	Australia	Parents with children	304	Mixed-methods design (evaluation study)	Not specific (vaccine-preventable childhood diseases)	Attitudes towards vaccine (−)
Encourage emotional affect	Papapchrisanthou and Loman (2018; *Public Health Nursing*)^86^	USA	Parents of infants aged 4–14 days	40	Non-randomised study	DTaP, Polio, Influenza, Hepatitis B and Pneumonia	Parental perception of immunisations, perceived knowledge of disease, comfort with immunisation decision making, satisfaction with provider and vaccine schedule adherence (0)
	Blanchard *et al* (2020, *Journal of Public Health*)^87^	USA	Adolescents aged 13–18 years	598	Non-randomised study (single group pre-test and post-test)	Not specific (vaccine-preventable childhood diseases)	Knowledge, attitudes and beliefs about vaccines (+)
	Kuru *et al* (2021, *PLoS On*e)^88^	USA	Adults	2345	RCT	MMR	Vaccine intentions (MMR vaccines) (+)
	Nyhan *et al* (2014, *Pediatrics*)^91^	USA	Parents with children aged <17 years	2229	RCT	MMR	Perception about vaccine’s safety and intention to vaccinate (MMR vaccine) (−)
	Kepka *et al* (2011, *Journal of Community Health*)^89^	USA	Parents with daughters aged 9–17 years	88	RCT	HPV	Vaccination awareness and uptake (HPV) (+)
	Cox *et al* (2010, *Health Psychology*)^90^	USA	Mothers with daughters aged 11–16 years	522	RCT	HPV	Intention to vaccinate (HPV) (+)
Change defaults	Giubilini *et al* (2019, *HEC Forum*)^73^	UK	General population (61.9% are parents with children)	457	Quantitative descriptive study (survey)	MMR	Vaccination policy at schools (+)
	Reiter *et al* (2012, *Journal of Behavioural Medicine*)^74^	USA	Parents with adolescent sons aged 11–17 years	404	RCT	HPV, influenza and meningococcemia	Intentions to vaccinate (HPV, seasonal influenza and meningococcal vaccine) (0)
	Brewer *et al* (2017, *Pediatrics*)^72^	USA	Primary care clinics with patients aged 11 or 12 years	29	RCT	HPV	Vaccination uptake (HPV vaccine) (+)
	Opel *et al* (2015, *American Journal of Public Health*)^71^	USA	Paediatric providers and parents of children aged 1–19 months	16	Non-randomised (cross-sectional observational study)	Not specific (vaccine-preventable childhood diseases)	Parental verbal acceptance of recommended vaccines at visit’s end (0)
Offer incentives	Zeng *et al* (2019, *Vaccine*)^67^	China	Parents with young children aged 3–6 years	1506	Non-randomised (cross-sectional survey)	Influenza	Intention to vaccinate (influenza vaccine) (+)
	Buttenheim *et al* (2016, *Vaccine*)^45^‡	USA	Parents and caregivers of infants	95	RCT (feasibility study)	Tdap	Vaccination uptake (Tdap) (−)
	Bronchetti *et al* (2015, *Journal of Economic Behaviour and Organisation*)^68^	USA	College students	9358	RCT	Influenza	Intention to vaccinate (influenza vaccine) (0)
	Rockliffe *et al* (2020, *PLoS One*)^69^	UK	Students aged 13–14 years	36 students (n=6 FGDs), 181 students	Qualitative design	HPV	Acceptability of financial incentives (0)
	Banerjee *et al* (2010, *BMJ*)^70^	India	Households with children 0–5 years	2188	RCT	Not specific (vaccine-preventable childhood diseases)	Vaccination uptake (all childhood vaccines) (+)
Change the messenger	Schoeppe *et al* (2017, *Health Promotion Practice*)^75^	USA	Parent advocates	33 (across three study years)	Mixed-methods design (evaluation study)	Not specific (vaccine-preventable childhood diseases)	Knowledge, attitudes and behaviour on vaccines (+)

*The sign in the brackets indicates whether the results support the effectiveness of the respective intervention (+), indicate negative evidence for the effectiveness (−) or the results are mixed (0).

† Used an additional nudge - ‘Change the messenger’

‡ Used an additional nudge - ‘Invoke social norms’

DTaP, diptheria, tetanus and accelular pertussis; FGD, focus group discussion; MMR, measles, mumps and rubella; PCV13, pneumococcal conjugate; RCT, randomised controlled trial; Tdap, tetanus, diphtheria and pertussis.

### Quality of evidence in included studies

According to the MMAT-based scoring (online supplemental tables 2–6), all included studies had clear research questions, data collection procedures, and a study design suitable to achieve the aims and objectives. However, some studies lacked explanations regarding how the sample size was determined. Mixed-methods studies sometimes demonstrated a lack of coherence between the qualitative and quantitative components, especially regarding the joint analysis of the main outcomes. Quality scores were relatively similar within study design groups. No study was eliminated because of a low MMAT score.

According to the GRADE scoring of the 28 RCTs included in the review (online supplemental table 7), nine studies were ranked as high quality, whereas six studies were graded as very low quality. The presence of biases (ie, information and selection), lack of clear blinding procedures, large losses to follow-up, and unclear or missing sample size calculations were among the most common reasons for quality score downgrades.

### Nudging interventions: descriptions and timing

Drawing on the MINDSPACE framework and the nudging literature,^41–43^ we identified a total of seven types of nudging interventions employed across the selected studies (table 3). Intervention delivery varied across studies depending on the type of nudging technique employed, target population, as well as the timing of intervention delivery (eg, postpartum) and duration of intervention exposure. We present our results in descending order of strength of evidence rather than following the lettering of the acronym MINDSPACE. In cases when a study drew on multiple nudges (n=2),^44 45^ we describe the study’s findings in multiple nudge subsections.

**Table 3:** Summary of nudging interventions derived from this systematic review

Type of nudging	Rationale	Nudging-based approaches employed in the included studies
Make available information salient	People behave differently, depending on which information is salient in that particular moment.^41^	Reminders (vaccinations due soon) and recalls (vaccinations past due) increase coverage across populations, vaccines and mode of delivery: use of personalised celebration card sent to parents/caregivers before their children’s vaccination schedule;^46^ directly sending individualised letters (in a coloured banner with aboriginal artwork and highlighting the word ‘free’)^47^ or reminders via personalised letters, postcards or text message to increase vaccination uptake;^47–52^ use of computer-based and phone-based software programs to generate automatic reminder text messages to parents;^53 54^ use of an electronic health record-based decision support system for clinicians to receive an automated reminder to recommend initial or succeeding doses of vaccines to patients;^55–57^ use of a familiar and trusted source (eg, own physician vs the national health system) to deliver vaccination reminders/recall;^60 61^ use of improved screening tools and automatic provider notification or reminders;^58^ use of an email prompt to spark vaccination intention among employees;^59^ use of motivational interviewing phone calls to parents to address commonly asked questions about vaccine.^48^
		An invitation that recommends getting an early shot to increase vaccine effectiveness.^66^
		Emphasis on disease salience, disease threat and promotion of self-efficacy to increase intention to vaccinate via educational messages, videos or pamphlets among pregnant mothers and parents.^62–65^
Offer incentives	Incentives, both tangible and intangible, can increase favoured behaviour.^41 42^	Offering incentives for vaccinations: giving vouchers for free or discounted vaccines to increase uptake;^45 67 68^ offering parents 1 kg of raw legumes per immunisation schedule and a set of meal plates after completion of immunisation schedule.^70^
		Offering incentives to promote return of vaccine consent forms among adolescents.^69^
Change defaults	Changing whether something is the default option versus requiring explicit opt-in can change behaviour.^41 42^	Presenting parents with hypothetical options to *opt in* (vaccination would only occur if parents completed a form saying to) or to *opt out* (vaccination would occur unless parents completed a form saying not to) of vaccinations.^73 74^
		In the doctor–patient interaction, switching to presumptive communication (eg, ‘Today we are vaccinating your child.’) from participatory communication (eg, ‘Will we do vaccinations today?’).^71 72^
Change the messenger	Changing who delivers the message (eg, a trusted source or a peer) can alter how the message is received.^41 43^	Training parental advocates on vaccination to address the issue in their peer group and community.^75^
		Delivering provaccine messages by ordinary people as opposed to medical experts.^44^
Change the way outcomes are framed	The same outcome is perceived differently when communicated in terms of benefits gained (eg, lives saved) versus losses avoided (eg, deaths averted).^41 43^	Framing vaccinations in terms of gain-framed messaging (describing benefits of receiving vaccines) versus loss-framed messaging (describing the cost of not receiving the vaccine);^76 77 79–81^ vaccine benefits on an individual rather than societal level;^44 78 82^ message order (from positive (vaccine effectiveness) to negative (vaccine side effects) or vice versa).^83^
Invoke social norms	Being aware of how others behave in a particular situation or feeling strongly about an issue can cause people to change their own behaviour.^41 43^	Using a storytelling narrative video to tell the parents that their community members, friends and doctors think that they should be vaccinated.^84^
		Using community campaigns and parent advocates to explicitly appeal to local values around social justice, parenting and alternative lifestyles.^85^
		Featuring a celebrity to establish a social norm about vaccination.^45^
Encourage Emotional Affect	The use of emotional associations (words, images and events) affects behaviour.^41^	Using visually enhanced education (images, videos, personal experiences and anecdotes) to elicit emotional connections to increase perception of vaccine effectiveness and comfort.^86–88^
		Using dramatic narratives: featuring an infant who almost died of measles and displaying visuals or ‘disease images’^91^ embedding narratives in radio programming to stimulate a more realistic experience.^89^
		Graphical presentation of vaccine risk statistics accompanied by a rhetorical question such as ‘Do you want to protect your daughter? If there was a vaccine to protect your daughter against cancer, would you have her get it?’.^90^

#### Make available information salient

Nearly half of the included studies (21 of 48) used different ways of salient messaging (eg, novel, accessible and simple information) to capture attention and directly relate it to the target population’s personal experiences to promote vaccination behaviours.

A qualitative study in the UK assessed the feasibility and acceptability of a personalised ‘celebration card’ (codesigned with parents/caregivers) to accompany the child’s vaccination record, alongside an information leaflet preceding the vaccination schedule.^46^ Parents perceived the intervention as an effective way to prompt them to take the necessary action.^46^

An RCT in Australia evaluated the use of individualised letters (with Aboriginal artwork and information on vaccines highlighting the word ‘FREE’ to indicate they were free of cost) and pamphlets (with photographs of Aboriginal families and the same informational material) among Aboriginal Australians.^47^ The findings showed that the letter intervention resulted in significantly higher influenza vaccination rates compared with the control and the pamphlet groups.^47^ A study in the USA evaluated the use of reminders via postcards and text messages, including the conduct of motivational interviewing phone calls guided by a licensed clinical psychologist, which proved feasible and acceptable to parents who were targeted with HPV vaccine reminder messages.^48^ The results echoed findings in another study in the USA that used 19 different text-based nudges (varied based on timing and contents of the messages) developed by behavioural scientists and designed to increase influenza vaccine uptake.^49^ Overall, results showed a statistically significant boost in vaccination across the nudges, but the top-performing nudge was a reminder that said ‘an influenza vaccine has been reserved for you’, which was associated with an 11% increase in vaccination uptake.^49^ A mixed-methods study in Italy demonstrated that a personalised individual letter significantly increases influenza vaccine intentions among healthcare workers (HCWs) who received the nudge intervention compared with the control group.^50^ An RCT in the USA showed that sending reminder postcards to elderly adults who were late for at least one vaccine significantly increases vaccination rates.^51^ On the other hand, using the same approach in terms of frequency and nature of reminders, an RCT in a large acute care hospital in the UK found no evidence that influenza vaccine uptake is affected by reminder letters.^52^

In Bangladesh, an RCT demonstrated that the use of an application called ‘mTika’ to automatically generate reminder messages for mothers in hard-to-reach rural and urban areas was effective in improving vaccination coverage.^53^ However, a similar approach in an RCT of 90 parents of newborns in the USA found that the automated reminders 7 days before babies’ next scheduled vaccination (at 2, 4 and 6 months of age) did not increase uptake.^54^ On the other hand, an observational study also in the USA evaluated the use of active choice (ie, a method that requires providers to accept or decline vaccination orders) through electronic health records, which demonstrated a substantial increase in adult influenza vaccination rates compared with the control group over time (>2 years observation period).^55^ Two studies in the USA using a comparable approach among patients of primary clinics showed a significant increase in influenza vaccination uptake over a 2-year to 3-year period compared with the control groups.^56 57^

Automatic notifications were found to be more successful among vaccine providers. Duvall,^58^ for example, developed a collaborative approach (integrating nursing, pharmacy, providers and information technology) to improve the process of ordering influenza vaccines for patients. Physicians and nurses were trained to become ‘champions’ on influenza vaccine screening and administration. Results showed that combining these automatic provider notifications with a vaccine-screening tool significantly increased influenza vaccination uptake. An RCT in the USA designed an email prompt to bolster influenza vaccination rates among 3272 employees (≥50 years old) of a large utility firm.^59^ Participants were randomly assigned to receive personalised email prompts; employees who received a prompt around a date and time they planned to be vaccinated had a significant increase in vaccine uptake compared with the control group.^59^

Also, in the USA, findings from an RCT compared the impact of mailing postcards (directly mailed by the national immunisation information system to families) with practice-based reminders (HCWs delivered the postcards).^60^ Directly mailed postcards achieved a significant increase in vaccination rates, whereas only 5% of reminders were delivered to families in the practice-based approach, suggesting (the study’s authors argue) that a high workload in primary care facilities can affect the implementation of facility-based reminders.^60^ Similar findings in an RCT in the USA demonstrated a small although significant effect among intervention groups on influenza vaccine uptake compared with control groups that received no reminders.^61^ These findings suggest that technological interfaces can be a central resource to support health facilities experiencing difficulties in efforts to remind parents of vaccination schedules.

An RCT conducted in Japan used a stepwise vaccination education programme with a focus on salient messaging for mothers, which entailed delivery of educational leaflets. The messaging approach began before giving birth until 3–4 days postpartum and 1 month after delivery to assess the infant vaccination uptake.^62^ While the results showed no difference in *Haemophilus influenzae* type b (Hib) and pneumococcal conjugate (PCV13) vaccine uptake, the findings revealed an increase in parents’ perception of the benefits of vaccines, viewing vaccines as a component of motherhood.^62^ In the USA, three studies that sought to use tailored messages to improve HPV vaccine uptake specifically demonstrated no intervention effects.^63–65^ While one of the US studies showed increase in participants’ knowledge about HPV vaccines,^64^ others did not.^63 65^

Based on an RCT conducted in Israel, an invitation (‘getting early shot to increase vaccine effectiveness’) combined with traditional information approaches about stock (‘vaccine is more likely to run out of stock’), benefits (‘early shot carries monetary benefits’) and costs (‘early shot is free while the late costs a fee’) is effective compared with the intentions of getting influenza shots late.^66^

#### Offer incentives

Five studies used incentives as a mechanism to motivate behaviour change. Recognising that the cost of vaccines is a key barrier to uptake in China, a cross-sectional survey compared a free policy and non-free policy of influenza vaccination among parents of young children and found that influenza vaccination coverage was higher with the free policy (34.2%) compared with the non-free policy (3.1%).^67^ In the USA, a study assessed the feasibility of delivering vouchers (for free and discounted vaccines) to parents of infants to increase pertussis vaccination uptake.^45^ The study also combined the experimental use of vouchers with a video on the importance of the Tdap (tetanus, diphtheria, pertussis) vaccine featuring Jennifer Lopez (a celebrity) as a means to increase salience and establish social norms about vaccination. Although the results indicated an increase of awareness regarding pertussis risk, vaccine uptake remained very low across the four-arm intervention design.^45^ Also in the USA, an RCT examined the use of a routine email highlighting monetary incentives to vaccinate among college students.^68^ The findings suggest a significant increase in influenza vaccine intentions among students who received a message about a financial incentive (email subject line: ‘Influenza vaccine (get $30)’), compared with the control group.^68^ A different approach was performed in the UK, wherein participants were incentivised via a lottery to return informed consent forms; regardless of whether forms included or withheld signatures, winning participants could receive US$50/€60 in a prize draw.^69^ Qualitatively, participants in the consent raffle described feeling generally good about the intervention as a whole, but several questioned the ethics of incentivising vaccine-related behaviours as this could be too akin to bribery.^69^

In rural India, an RCT evaluated vaccination campaigns with and without incentives.^70^ A control group was compared with groups that included (1) an ‘immunisation camp’ (where immunisation services were provided by a nurse and an assistant on a fixed schedule); and (2) an ‘immunisation camp plus’ model that also included a non-financial incentive (1 kg of raw lentils per vaccination conducted on schedule and a set of metal plates on completion of all childhood vaccines).^70^ Parents who received non-financial incentives were more likely to complete vaccination compared with controls, as long as vaccines were reliably available at respective health facilities.^70^

#### Change defaults

Setting defaults has also been used with regard to vaccination-related patient–provider communication. A cross-sectional study in the USA used participatory (built on the principle of shared decision making) and presumptive (presupposing those parents would decide to vaccinate their child the same day) approaches to increase vaccination acceptance. The presumptive format was associated with an increase in parental vaccine acceptance but also with reduced satisfaction in the clinical experience, while the participatory format showed the opposite pattern.^71^ An RCT in the USA supported these results in a study that examined the effectiveness of announcements (presumptive) and conversations (participatory) as compared with the usual care for increasing HPV vaccination uptake in 30 primary care clinics with 100 or more patients aged 11–12 years.^72^ Their findings also indicated an increased vaccination uptake among those randomised to the announcement group; no statistical difference was noted among the conversation group compared with the control group.^72^

A survey in the UK investigated the use of defaults to increase catch-up vaccines (ie, for those who missed or did not complete a vaccine regimen) on measles, mumps and rubella (MMR) among members of the general population (62% of whom are parents with children).^73^ Researchers sought participants’ opinions regarding five possible policies for secondary schools: (1) no MMR catch-up vaccination at school, (2) catch-up MMR vaccination with explicit parental consent (3) MMR catch-up vaccination offered based on general parental permission at school enrolment, (4) vaccination voluntary but permission generally assumed unless parents explicitly indicate otherwise (opt-out) and (5) mandatory MMR catch-up vaccinations at secondary schools.^73^ Participants were most favourable of opt-out and general permission policies, and highly opposed to the mandatory and no vaccination policies.

An RCT in the USA presented parents with a hypothetical scenario of moving to a new state where their adolescent son is starting at a new school.^74^ Participants were randomly assigned to one of three groups: (1) the new school had an opt-in vaccination policy (new students being vaccinated only if parents consented), or (2) an opt-out vaccination policy (vaccination occurs by default unless parents object) or (3) a neutral condition where parents had to complete a form whether or not their child would be vaccinated. Afterwards, parents were asked about their intention to vaccinate against HPV only, or HPV vaccine along with seasonal influenza and meningococcal vaccine. Results showed that those parents in the opt-in policy group were more likely to vaccinate compared with the opt-out group, contradicting common assumptions with regard to the power of defaults.^74^ Additionally, there was no effect of different defaults among parents who were undecided as to whether their adolescent son should receive the vaccine, suggesting that strong, conflicting feelings about the HPV vaccine in general might hinder the effect of default policies on vaccination uptake.^74^

#### Change the messenger

Two studies focused their nudging intervention on changing the messenger assigned with communicating vaccine-related health information.^44 75^ In the USA, a mixed-methods study used a community approach (‘immunity community’) to shift the perceived authority to parents themselves, aiming to build a stronger connection among peers.^75^ The intervention mobilised parent advocates to establish dialogues about vaccines through approaches such as one-on-one communication with peers, social media advocacy, events and distribution of immunisation educational materials.^75^ The results showed statistically significant improvements in parental knowledge and attitudes towards vaccines, but parental vaccine-related behaviours did not change significantly.^75^

#### Change the way outcomes are framed

A non-RCT in Ireland presented 72 parents of young girls with a one-page summary describing risks of an HPV infection. Parents were then randomly assigned to either receive a gain-framed message (describing the benefits of getting the vaccine) or a loss-framed message (describing the risks of not getting the vaccine).^76^ The findings suggest a strong intention to vaccinate against HPV regardless of the framed messages but found no significant effect of framing in vaccination attitudes, normative beliefs or perceived behavioural control.^76^

In the USA, a mixed-methods study investigated the effects of different ways of framing vaccine-related information on HPV vaccine intentions among three cultural groups (n=150, 50 per ethnic group) (Hispanic, non-Hispanic white, and non-Hispanic African-American).^77^ The results revealed significant differences: for the Hispanic group, both frames equally increased vaccine intentions, while for the other two study groups, a loss frame message was more effective in increasing intentions to vaccinate. An RCT in the USA, conducted prior to the roll-out of COVID-19 vaccines, found that intentions to vaccinate against COVID-19 increased significantly when the messages are framed towards personal health risks, consequences of not getting the vaccine, and if these framed messages come from medical experts.^44^ In a similar approach, in the UK, an RCT demonstrated that the use of personal benefit messages was more effective among individuals who are strongly hesitant about COVID-19 vaccines compared with those who received information on collective benefits.^78^

In China, a study examined women’s (aged 18–45 years old) intention to vaccinate their children (and future children) with HPV vaccines using three types of messages: (1) gain-framed (lower chance of contracting cervical cancer and genital warts), (2) loss-framed (higher chance of contracting cervical cancer and genital warts), and (3) narrative (participants were presented with a story of a young mother related to HPV and HPV vaccine).^79^ Vaccine intentions did not vary significantly by group; however, gain-framed messages seemed to appeal more to future-minded women, and narrative messages appealed more to present-minded women.^79^ On the other hand, another study in China demonstrated that the loss-framed messages lead to a more favourable intention towards HPV vaccines as opposed to the gain-framed messages.^80^ Using a similar approach, a longitudinal study among minority pregnant women in the USA showed no significant difference of either gain-framed or loss-framed messages on influencing maternal vaccination rates.^81^

Employing a different framing approach in the USA, an RCT demonstrated that framing vaccine benefits on an individual level (the vaccine recipient) significantly increased vaccination intentions compared with the same information being framed in terms of societal benefits.^82^ Meanwhile, another RCT trialled the use of ‘positive–negative message order’ (information on vaccine effectiveness presented first, followed by vaccine side effects) and compared it to ‘negative–positive message order’ combined with message calendar (with or without plans of getting the vaccine).^83^ However, the findings found no statistically significant effects of message order on vaccine intentions.^83^

#### Invoke social norms

Three studies highlighted the use of social norms (eg, normalising vaccination as a socially acceptable behaviour or featuring a celebrity to establish a norm) in existing social networks (eg, network members, friends, family and healthcare providers) to shape their knowledge and intention to vaccinate.^45 84 85^ A pilot RCT among Cambodian–Americans in the USA found that the use of a storytelling narrative video could increase engagement and persuasion.^84^ The video showed Khmer mothers and Khmer physicians discussing issues around getting vaccinated, eventually normalising vaccines and depicting vaccination as a desirable behaviour that benefits the entire community.^84^ Results showed higher intention to vaccinate in the intervention group versus the control group, which only received educational flyers on the HPV vaccine, but the study focused on feasibility, and vaccine uptake was not evaluated.^84^ The acceptability of the intervention was relatively high, and participants reported an emotional connection and relatability to the images and stories.^84^

On the other hand, a mixed-methods study in Australia concluded that using a values-based approach can in fact polarise vaccination attitudes and therefore be counterproductive.^85^ The ‘I Immunise’ campaign used community advocates and featured their alternative lifestyle (attributes including home birthing, breast feeding, baby-wearing wraps and cloth nappies, and eating wholefoods) in a photograph combined with testimonial of how vaccines were a part of their alternative lifestyle.^85^ Results, however, did not support this novel approach: some participants expressed more negative attitudes regarding vaccination postcampaign as compared with baseline, and the campaign led to complaints that the presented material was one-sided and could reinforce stereotypes regarding lifestyle and vaccine decisions.^85^

#### Encourage emotional affects

Six studies employed dramatic narratives and graphics in the context of vaccine messaging. A non-RCT in the USA found that using pictures of patients with vaccine-preventable diseases could improve parental perception about vaccines and vaccine schedule adherence.^86^ Parents of infants in the intervention group demonstrated increased vaccination knowledge and were more satisfied with their providers compared with those parents who received no intervention, although changes in the perception of vaccine effectiveness and comfort with decision making was not statistically significant. Another non-RCT conducted in the USA demonstrated that the use of graphical images, videos and interactive scenarios can substantially improve high school students’ knowledge, attitudes and beliefs in vaccines.^87^ Also, in the USA, an RCT was conducted that used five different videos ranging from personal stories to statistical or narrative science-supporting messages.^88^ The findings suggest that science-supporting messages (ie, a video with Dr. Anthony Fauci, Director of National Institute of Allergy and Infectious Diseases, providing statistical information about the contagiousness of measles, vaccine safety and vaccine effectiveness) influence provaccine views and vaccine intentions among American adults.^88^

An RCT in the USA found that a vaccination-related *radionovela* (a dramatic story broadcast on the radio) of a young girl’s journey of hearing about and ultimately receiving the HPV vaccine improved knowledge and attitudes about vaccines among US Hispanic parents or guardians of young children.^89^ Furthermore, another RCT, also in the USA, found that showing mothers a presentation on cancer prevalence and lives saved by the vaccine, combined with rhetorical questions (such as ‘Do you want to protect your daughter from cancer?’), could increase message comprehension and had an overall positive effect on mothers’ intention to vaccinate, versus a non-graphical presentation and a control group.^90^

In the USA, Nyhan and colleagues^91^ used ‘disease images’ and dramatic narratives of an infant who almost died of measles to portray the risk of not vaccinating to parents with children under 17, and found no significant positive effects of these provaccine messages. Instead, they highlighted that using emotionalised stories designed to instil fear could increase vaccine safety concerns among those who were already hesitant to vaccinate.

### Effect of nudging interventions

A variety of outcome measures were used to assess the effect of nudging interventions. The most frequent were vaccination uptake, intention to vaccinate, parental knowledge and attitudes towards childhood vaccinations, and vaccination acceptability and satisfaction. To better illustrate the results across approaches, we plotted the results on a matrix (‘harvest plot’^92^) to summarise and compare the evidence of individual studies (see table 4). Each of the three table columns lists studies that disfavour (no significant effect) or favour (significant effect) nudges, or that present mixed results. Each study is presented as a bar highlighting certainty of evidence; darker shaded bars are RCTs (graded on a 4-point GRADE scale) and lighter shades represent non-RCTs and other study designs (graded on a 5-point MMAT scale) (see online supplemental tables 2–7). Higher bars represent lower risk of study bias. Finally, numbers within each bar refer to study references as cited in the reference list.

**Table 4:** Harvest plot of nudging outcomes among the general population included in this systematic review*

	Disfavours nudges 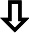	Mixed results 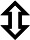	Favours nudges 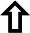
**Feasibility, acceptability, & satisfaction**			
Make available information *salient*	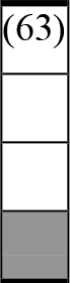		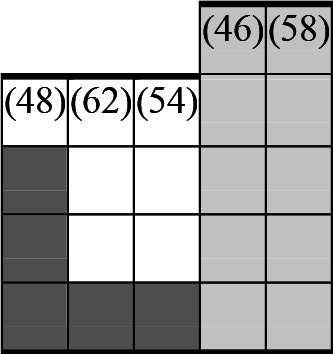
Change the way outcomes are *framed*	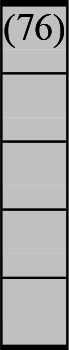		
Invoke *social norms*	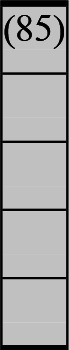		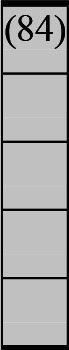
Encourage *emotional affect*	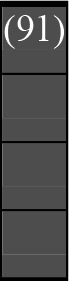		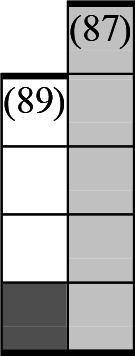
Change *defaults*		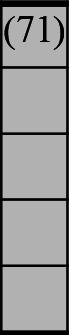	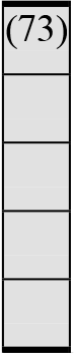
Offer *incentive*		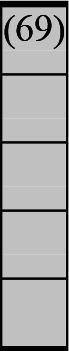	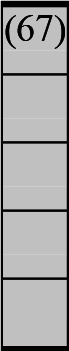
**Knowledge & awareness about vaccination**			
Make available information *salient*	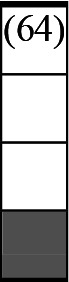		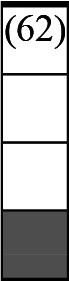
Change the way outcomes are *framed*	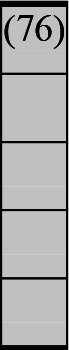		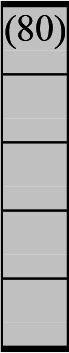
Encourage *emotional affect*	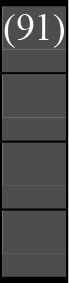	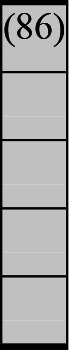	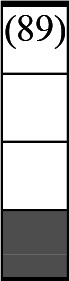
Offer *incentive*			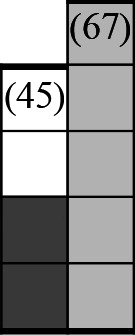
Change the *messenger*			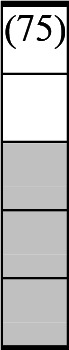
**Intention to vaccinate**			
Make available information *salient*	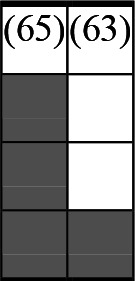		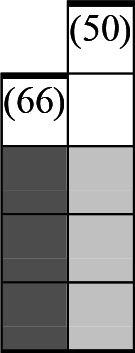
Change the way outcomes are *framed*	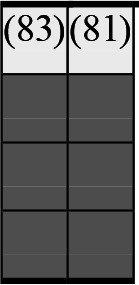	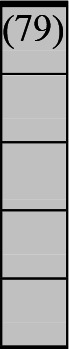	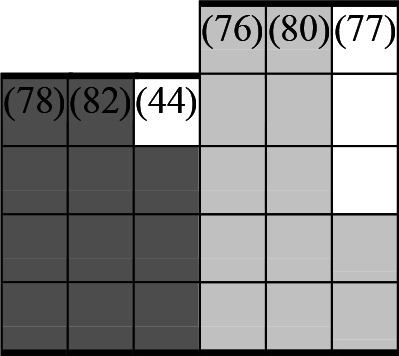
Invoke *social norms*			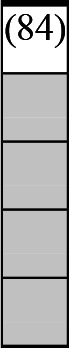
Encourage *emotional affect*	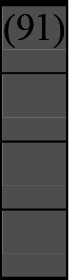		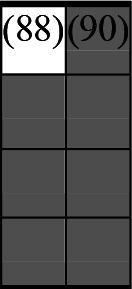
Change *defaults*		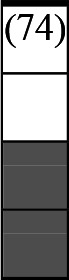	
Offer *incentive*	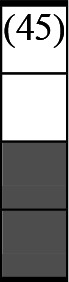	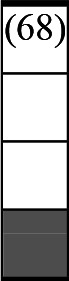	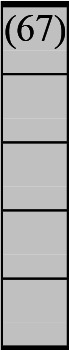
**Vaccination uptake**			
Make available information *salient*	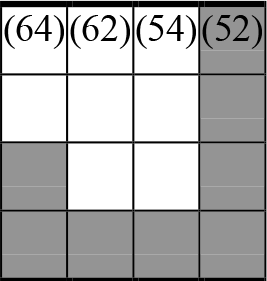	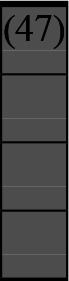	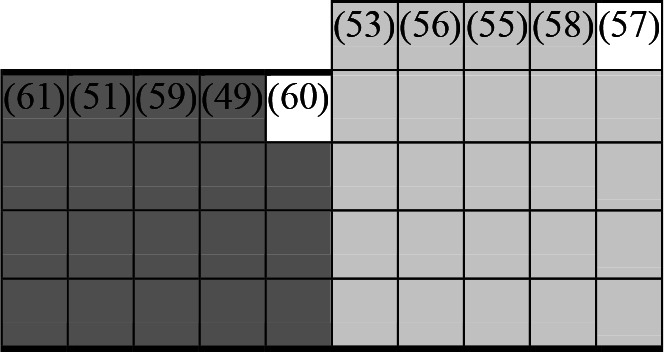
Change *defaults*		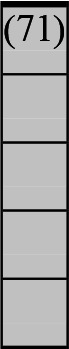	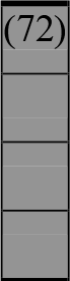
Offer *incentive*	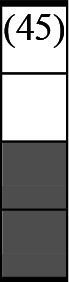		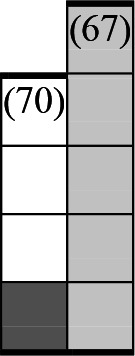

* Each study is presented as a bar highlighting certainty of evidence; darker shaded bars are RCTs (graded on a 4-point GRADE scale) and lighter shades represent non-RCTs and other study designs (graded on a 5-point MMAT scale). Numbers within each bar refer to study references as cited in the reference list.

In a majority of studies assessing how participants perceived a given nudging intervention (10 of 16), participants reported that the approach was acceptable, feasible and satisfactory. In 6 of the 10 studies that assessed vaccination knowledge and awareness, the nudge of providing incentives, saliency of information, framing messages, encouraging emotional affect and changing the messenger were reported to have a positive effect. Among the 19 studies that looked at vaccination uptake as the primary outcome of interest, 12 studies found statistically significant increases in vaccination coverage compared with control groups. Roughly half (12 of 21 studies) showed a positive effect on vaccination intention among study participants.

## Discussion

This systematic review is an up-to-date synthesis of available evidence on how nudging affects intentions and behaviours related to vaccines and vaccine uptake, thus providing important guidance related to the ongoing COVID-19 pandemic and beyond. We identify a number of nudging approaches, including some codeveloped by scientists, community members and HCWs, several of which have shown promising results in changing vaccination attitudes or behaviours. Arguably, the most promising evidence in terms of vaccine uptake exists for nudging interventions that make available information more salient, offer incentives to parents and HCWs, and change defaults. While some studies seem to be efficacious in increasing vaccine uptake, only five included studies were designed within a controlled experimental setting.^49 51 59–61^ Of these studies, nudges such as ‘use of reminders and recalls’, ‘providing tangible and intangible incentives’ and the ‘use of emotional associations (eg, videos and images)’ increased favoured behaviours towards vaccines. Additionally, our findings highlight that a majority of studies have assessed the impact of nudging on vaccination attitudes and intentions instead of observed vaccination behaviour. While we acknowledge the challenges of assessing actual vaccination uptake as a result of nudging interventions, approximating it via attitudes and intentions holds risk of biases, especially in light of studies highlighting that the effect of nudges tends to be less long-lived.^24^

Some of the identified nudging interventions are driven by emotional appeals using salient messages, with evidence showing they could increase vaccination intentions and uptake.^53 55 56 58 60^ However, one must be cautious and provide a targeted nudging approach only to those parents who are ‘fence sitters’ with concrete doubts or questions regarding vaccines.^74 85^ Some studies suggest that using nudging interventions with those groups already experiencing strong and conflicting feelings towards vaccination can backfire.^74 85^ Indeed, Smith and colleagues^93^ discuss the influence of heuristics and beliefs in building vaccine confidence, highlighting how facts contradicting previously held beliefs are often difficult to accept. Screening parents to ascertain their views on vaccination before nudging may be a viable option and is likely a critical step prior to implementation of any vaccine nudge intervention.

Several studies showed evidence of the role of the ‘antivaxxers’ movement in the sudden decline of vaccine confidence and uptake of childhood vaccinations.^15 94 95^ These groups are relatively small in number, but their internet reach and coverage are extensive.^96^ Several scholars have outlined ways to counter antivaccination groups and address VH by developing provaccine messages rooted in narratives and social norms, using visually enhanced education materials and interventions that are focused on regaining trust.^97–100^ However, Attwell and Freeman^85^ found that while their value-based approach gained large amounts of social media mileage, efforts redoubled from antivaccination groups to warn communities of side effects of the vaccines, and to attack the material as one-sided and stereotypical. This highlights how the antivaccination community must be considered when promoting a new intervention, especially on platforms and social media with a history of a highly emotionalised vaccine-related discourse (eg, Facebook).^96 101^ Similarly, emotionalised stories and shocking images designed to inflict fear can increase vaccine safety concerns among parents.^91^

Several LMICs are currently experiencing sudden declines of vaccine confidence,^102^ a development which is particularly alarming as the risks of an outbreak of a vaccine-preventable disease for individuals and health systems are disproportionally larger in resource-poor settings.^17 103 104^ While our findings hold relevance for countries across the globe, nudging interventions are often considered to be less costly than many other large-scale health intervention programmes,^41 105 106^ highlighting their potential for LMICs or other resource-limited settings. We also found that a nudging approach that is implemented successfully in one setting cannot always be successfully transferred to another setting or another group of people, underscoring the importance of design research to determine how to best adapt and trial optimal nudges.^107^

Several studies highlighted ethical concerns, including that nudging restricts individual freedom of choice^73 108^ and promotes coercion of parents and HCWs.^109–111^ Thaler and Sunstein^112^ refer to ‘libertarian paternalism’ as an approach that preserves and promotes autonomy but allows authorities to direct individuals in a positive direction by providing options that are subtly more or less accessible. However, a number of authors have highlighted the importance of distinguishing between nudges and shoves.^113 114^ Shoves, as opposed to the nudges described in this paper, use a much more directive approach, as described in the ‘ladder of interventions’ of the Nuffield Council on Bioethics,^113^ such as in the ‘no jab, no pay’ policy in Australia, denying unvaccinated children access to public places in the USA, or charging parents of unvaccinated children substantial fines in Germany.^109–111^

Health systems in general and vaccination challenges in particular are complex and generally not resolved through single interventions at the family or individual levels alone. Instead, multipronged approaches including communities, hospitals or points of care, and policymakers are needed and essential.^115^ A number of scholars around the world have demonstrated that there is no ‘one-size-fits-all’ approach for nudges to promote vaccination uptake and address VH.^116–118^ This is particularly true for LMICs with unique patterns of VH and structural conditions such as unreliable availability of vaccines,^17^ and where relevant scientific evidence remains limited.

VH is an urgent public health problem not only in the context of already existing vaccines with well-established efficacy and safety, but also with regard to novel vaccines, where limited uptake by the population could have disastrous implications.^119^ With COVID-19 vaccines currently being rolled out in many countries, several studies already highlighted concerns that acceptance of such a novel vaccine could be limited, with significant implications for vaccination programme effectiveness at the population level.^120 121^ The problem of VH requires exploring new pathways for how interventions, including nudges, can be implemented, including soon after vaccine introduction and in fluidly evolving contexts. In light of this need for novel approaches, our review also highlights that several nudging approaches (ie, the use of ‘priming’, ‘commitments’ and ‘ego’) which have shown promising results in other areas of health promotion, but have not yet been applied to VH and merit consideration.^41^ While a general review of nudges not yet applied to VH was beyond the scope of this paper, we do encourage further work on transferring otherwise successful nudging approaches to the field of vaccine confidence promotion.

### Recommendations

In light of our review, we present the following recommendations. For policymakers aiming at introducing VH interventions in their respective settings, we want to highlight the promising results of nudging-based interventions, particularly with regard to making available information salient, offering incentives, and changing defaults or the messenger. However, policymakers should be aware of the limited number of large-scale RCTs and the mixed evidence regarding the efficacy of the same nudge in different settings, especially with regard to LMICs. We therefore discourage transferring nudging interventions one-to-one to drastically different settings. Instead, a detailed understanding of the complexity of the target population, as well as comprehensive design research, is highly recommended to ensure the acceptability and effectiveness of the nudging intervention in the particular setting. Additionally, policymakers should look beyond vaccine recipients as intervention targets and consider broadening the scope to also include medical professionals and other stakeholders, and be aware of the risks of certain nudging interventions (eg, aimed at instilling fears) that may increase public doubt and provide additional arguments for antivaccination groups.^9 93^

With regard to future research, the gaps in terms of study design, sample size calculation and risk of biases highlight opportunities for more rigorous research to gauge the broader applicability of the nudging approach. Our results underscore that a relatively small number of studies have drawn on the concept of nudging regarding vaccination uptake, but promising results and the complexity of the concept, especially its applicability to different settings, call for further research. We also highlight a number of nudges which, to the best of our knowledge, have not yet been applied to VH but have shown promising results in other health promotion contexts, meriting further research. Furthermore, there is exceptionally limited information from LMICs informing this discourse, and qualitative research exploring acceptability regarding nudging interventions, both among policymakers and the general community, is similarly limited. The potential value and impact of nudging, and how nudges can profit from rigorous adaptation and acceptance from the community members are then paramount. We therefore urge scholars to consider these angles of investigation for future research.

### Strengths and limitations

The key strength of this systematic review is the rigorous search and identification of eligible studies across several databases using extensive search strings, overseen by a biomedical librarian (JL). The use of an iterative process at the outset of literature extraction ensured that we systematically considered all behavioural interventions and improved our chances of capturing nudging interventions. Nevertheless, some factors limit the generalisability of our results. The current small number of studies and the high variability with regard to quality, methods, measurement of VH, and outcomes across studies do not allow for a meaningful meta-analysis to ascertain its primary effects on vaccination uptake. The fact that most studies (46 of 48) took place in higher-income settings also conditions the interpretation of our findings, given the demonstrated variability of effects according to context. Also, we restricted this review to studies published since 2008, when the term nudging emerged.^25^ We note, however, that the concept of nudges as applied in healthcare predates this, and we encourage a more historical examination of nudges and other interventions to promote vaccinations. Finally, we excluded the term ‘immunization’ in the search criteria, and while we later identified and included several papers that employed this term following a hand screening of reviews, it is nevertheless possible that papers that employed the term ‘immunization’ rather than vaccination and were not in these reviews were missed.

## Conclusion

Nudging takes different forms and is delivered via different interventions. Early evidence suggests nudging may be a feasible and promising approach to increase public confidence in vaccines. Nudges targeting the vaccine recipients close to the decision whether or not to vaccinate (such as offering incentives, making more information salient, changing defaults and purposefully selecting the messenger) show the most promise. However, nudges targeting providers to facilitate vaccination uptake among their clients (eg, reminder systems) and therefore addressing VH from another angle are also valuable to consider and explore further. Successful public health programmes designed to increase vaccination uptake require context-specific adaptations to address target groups’ specific concerns and the understandings of vaccine-hesitant individuals. Further research, specifically in relation to nudging interventions in LMICs, is required.

10.1136/bmjgh-2021-006237.supp2 Supplementary data

## Data Availability

All data relevant to the study are included in the article or uploaded as supplementary information.
