# Supplementary material for: Nudging toward vaccination: a systematic review
Source: BMJ Glob Health. 2021 Sep 30;6(9):e006237. doi: 10.1136/bmjgh-2021-006237 (PMC8487203; doi:10.1136/bmjgh-2021-006237)
Supplement: Supplementary data [file bmjgh-2021-006237supp001.pdf]

Supplementary Files

Supplementary Table 1. Summary of research databases and search strings used in this review.		
Database		Search String
**1.**	PubMed	(((((((behavioral control) OR behavioral strategy) OR behavioral intervention)) OR ((nudging) OR nudge))) AND ((vaccination) OR vaccines))) AND (decision making))
**2.**	Science Direct	(“behavioral control” OR “behavioral intervention” OR “behavioral strategy” OR Nudding OR nudge) AND (vaccination OR vaccines) AND “decision making”
**3.**	ProQuest	((behavioral control) OR (behavioral intervention) OR (behavioral strategy) OR nudging OR nudge) AND (vaccination OR vaccine) AND (decision making)
**4.**	Ebscohost	(behavioral control OR behavioral intervention OR behavioral strategies OR Nudging OR nudge) AND (vaccination OR vaccine) AND (decision making)
**5.**	Oxford Journal	behavioral control OR behavioral intervention OR behavioral strategy OR nudging AND vaccination AND decision making
**6.**	Access Science	“behavioral control” OR “behavioral intervention” OR “behavioral strategy” OR nudging OR nudge AND vaccination OR vaccine AND “decision making”
**7.**	JSTOR	("behavioral control" OR "behavioral intervention" OR "behavioral strategy" OR nudging OR nudge) AND (vaccination OR vaccines) AND "decision making"
**8.**	Scopus	“behavioral control” OR “behavioral intervention” OR “behavioral strategy” OR nudging OR nudge AND vaccination OR vaccines AND “decision making”
**9.**	Google Scholar	“behavioral control” OR “behavioral intervention” OR “behavioral strategy” OR Nudging OR nudge AND vaccination OR vaccines AND "decision making"

Reñosa MDC, *et al. BMJ Global Health* 2021; 6:e006237. doi: 10.1136/bmjgh-2021-006237

**Supplementary Table 2. Quality assessment results for included RCTs, 2020.**					
**Included Study**	Randomized Controlled Trials				
	Q1	Q2	Q3	Q4	Q5
Borg, et al.**(47)**	1	1	1	0	1
Porter, et al.**(63)**	1	1	1	0	0
Saitoh, et al.**(62)**	1	1	1	0	1
Brewer, et al.**(72)**	1	1	1	1	1
Buttenheim, et al.**(45)**	1	1	1	1	0
Joseph, et al.**(64)**	1	1	1	0	1
Hendrix, et al.**(82)**	1	1	1	0	1
Nyhan, et al.**(91)**	1	1	1	1	1
Kempe, et al.**(60)**	1	1	1	0	1
Reiter, et al.**(74)**	1	1	1	0	1
Ahlers-Schmidt, et al.**(54)**	1	1	1	0	1
Kepka, et al.**(89)**	1	1	1	0	1
Banerjee, et al.**(70)**	1	1	1	0	1
Bronchetti, et al.**(68)**	0	1	1	0	0
Milkman, et al.**(59)**	1	1	1	1	1
Staras, et al.**(48)**	1	1	1	0	1
Milkman, et al.**(49)**	1	0	1	1	1
Szilagyi, et al.**(61)**	1	1	1	1	1
Okuno, et al.**(83)**	1	1	1	0	1
Motta, et al.**(44)**	1	1	1	0	1
Schmidtke, et al.**(52)**	1	1	1	0	1
Panozzo, et al.**(65)**	1	1	1	0	1
Chen, et al.**(51)**	1	1	1	1	1
Kuru, et al.**(88)**	1	1	1	0	1
Freeman, et al.**(78)**	1	1	1	1	1
Cox, et al.**(90)**	1	1	1	1	1
Frew, et al.**(81)**	1	1	1	0	1
Maltz and Sarid**(66)**	1	0	1	0	1

Notes:

Q1 - Is randomization appropriately performed? ; **Q2** - Are the groups comparable at baseline? ; **Q3** - Are there complete outcome data? ; **Q4** - Are outcome assessors blinded to the intervention provided? ; **Q5** - Did the participants adhere to the assigned intervention?

Scoring: Yes: 1 point; No: 0 point; Cannot Tell: 0 point.

Reñosa MDC, *et al. BMJ Global Health* 2021; 6:e006237. doi: 10.1136/bmjgh-2021-006237

Supplementary Table 3. Quality assessment results for included non-RCTs, 2020.

**Included Study**	Quantitative Non-Randomized Controlled Trials				
	Q1	Q2	Q3	Q4	Q5
Zeng, et al.**(67)**	1	1	1	1	1
Papapchrisanthou and Loman**(86)**	1	1	1	0	1
Uddin, et al.**(53)**	1	1	1	1	1
Opel, et al.**(71)**	1	1	1	1	1
Fahy and Desmond**(76)**	1	1	1	1	1
Patel, et al.**(55)**	1	1	1	1	1
Blanchard, et al.**(87)**	0	1	1	1	1
Xu, et al.**(80)**	1	0	1	1	1
Changolkar, et al.**(57)**	1	0	1	1	1

Notes:

Q1 - Is the sampling strategy relevant to address the research question? ; **Q2** – Is the sample representative of the target population?; **Q3** - Are there measurements appropriate?; **Q4** - Is the risk of nonresponse bias low?; **Q5** – Is the statistical analysis appropriate to answer the research question?

Scoring: Yes: 1 point; No: 0 point; Cannot Tell: 0 point.

Reñosa MDC, *et al. BMJ Global Health* 2021; 6:e006237. doi: 10.1136/bmjgh-2021-006237

Supplementary Table 4. Quality assessment results for included mixed-methods studies, 2020.

**Included Study**	Mixed-methods studies					Quantitative Studies					Qualitative studies				
	**Q1**	**Q2**	**Q3**	**Q4**	**Q5**	**Q1**	**Q2**	**Q3**	**Q4**	**Q5**	**Q1**	**Q2**	**Q3**	**Q4**	**Q5**
Lee, et al.**(84)**	1	1	1	0	1	0	1	0	0	1	1	1	1	0	0
Schoeppe, et al.**(75)**	1	1	1	0	0	1	1	1	1	1	1	1	1	1	1
Attwell and Freeman**(85)**	1	1	1	1	0	1	1	1	1	1	1	1	1	1	1
Lechuga, et al.**(77)**	1	0	0	1	0	1	1	1	1	1	1	0	0	1	1
Lorini, et al.**(50)**	1	0	1	0	1	1	1	1	1	1	1	1	1	1	1

Notes:

Mixed-methods: Q1 - Is there adequate rationale for using a mixed methods design to address the research question? ; **Q2** - Are the different components of the study effectively integrated to answer the research question?; **Q3** - Are the outputs of the integration of qualitative and quantitative components adequately interpreted?; **Q4** – Are divergences and inconsistencies between quantitative and qualitative results adequately addressed?; **Q5** – Do the different components of the study adhere to the quality of criteria of each tradition of the methods involved?

Quantitative studies: Q1 - Is the sampling strategy relevant to address the research question? ; **Q2** – Is the sample representative of the target population?; **Q3** - Are there measurements appropriate?; **Q4** - Is the risk of nonresponse bias low?; **Q5** – Is the statistical analysis appropriate to answer the research question?

Qualitative studies: Q1 Is the qualitative approach appropriate to answer the research question? ; **Q2** - Are the qualitative data collection methods adequate to address the research question?; **Q3** - Are the findings adequately derived from the data?; **Q4** - Is the interpretation of results sufficiently substantiated by data?; **Q5** – Is there coherence between qualitative data sources, collection, analysis and interpretation?

Scoring: Yes: 1 point; No: 0 point; Cannot Tell: 0 point.

Supplementary Table 5. Quality assessment results for included quantitative descriptive studies, 2020.

**Included Study**	Quantitative Descriptive Studies				
	Q1	Q2	Q3	Q4	Q5
Duvall**(58)**	1	1	1	1	1
Giubilini, et al.**(73)**	1	1	1	0	1
Liu, et al.**(79)**	1	1	1	1	1
Kim, et al.**(56)**	1	1	1	1	1

Notes:

Q1 - Is the sampling strategy relevant to address the research question? ; **Q2** – Is the sample representative of the target population?; **Q3** - Are there measurements appropriate?; **Q4** - Is the risk of nonresponse bias low?; **Q5** – Is the statistical analysis appropriate to answer the research question?

Scoring: Yes: 1 point; No: 0 point; Cannot Tell: 0 point.

**Supplementary Table 6. Quality assessment results for included qualitative studies, 2020.**					
**Included Study**	Qualitative Studies				
	Q1	Q2	Q3	Q4	Q5
Lwembe, et al.**(46)**	1	1	1	1	1
Rockliffe, et al.**(69)**	1	1	1	1	1

Notes:

Q1 - Is the qualitative approach appropriate to answer the research question? ; **Q2** - Are the qualitative data collection methods adequate to address the research question?; **Q3** - Are the findings adequately derived from the data?; **Q4** - Is the interpretation of results sufficiently substantiated by data?; **Q5** – Is there coherence between qualitative data sources, collection, analysis and interpretation?

Scoring: Yes: 1 point; No: 0 point; Cannot Tell: 0 point.

Reñosa MDC, *et al. BMJ Global Health* 2021; 6:e006237. doi: 10.1136/bmjgh-2021-006237

Supplementary Table 7. GRADE evidence profile for 25 randomized controlled trials included in this review.

Study	Quality assessment of evidence					
	Limitation	Inconsistency	Indirectness	Imprecision	Risk of bias	Quality grading
Borg, et al.**(47)**	No serious limitation	No serious inconsistency	No serious indirectness	No serious imprecision	None detected	⨁⨁⨁⨁ High
Porter, et al.**(63)**	Serious limitation (the trial was conducted via online, hence there is no confirmation if participants read the intervention)	No serious inconsistency	No serious indirectness	No serious imprecision	Very serious risk of bias (presence of information bias; large losses to follow-up)	⨁◯◯◯ Very low
Saitoh, et al.**(62)**	Serious limitation (there was an increased public awareness before the start of recruitment and participant enrollment that may have influenced study results)	No serious inconsistency	No serious indirectness	No serious imprecision	Very serious risk of bias (methods of blinding not mentioned; presence of information bias)	⨁◯◯◯ Very low
Brewer NT, et al.**(72)**	No serious limitation	No serious inconsistency	No serious indirectness	No serious imprecision	None detected	⨁⨁⨁⨁ High
Buttenheim, et al.**(45)**	Very serious limitation (no report of sample size calculation; no confirmation of utilization of intervention)	No serious inconsistency	No serious indirectness	No serious imprecision	None detected	⨁⨁◯◯ Low
Joseph, et al.**(64)**	No serious limitation	No serious inconsistency	No serious indirectness	Serious imprecision (sample size was not powered to detect significant difference)	Serious risk of bias (methods of blinding not mentioned)	⨁⨁◯◯ Low
Hendrix, et al.**(82)**	No serious limitation	No serious inconsistency	No serious indirectness	No serious imprecision	Serious risk of bias (methods of blinding not mentioned)	⨁⨁⨁◯ Moderate
Nyhan, et al.**(91)**	No serious limitation	No serious inconsistency	No serious indirectness	No serious imprecision	None detected	⨁⨁⨁⨁ High
Kempe, et al.**(60)**	No serious limitation	No serious inconsistency	No serious indirectness	No serious imprecision	Serious risk of bias (methods of blinding not mentioned)	⨁⨁⨁◯ Moderate
Reiter, et al.**(74)**	Serious limitation (sample size calculation was unclear)	No serious inconsistency	No serious indirectness	No serious imprecision	Serious risk of bias (methods of blinding not mentioned)	⨁⨁◯◯ Low
Ahlers-Schmidt, et al.**(54)**	Serious limitation (sample size calculation was unclear)	No serious inconsistency	No serious indirectness	Serious imprecision (sample size was not powered to	Serious risk of bias (large losses to follow-up; methods of	⨁◯◯◯ Very low

Supplementary Table 7. GRADE evidence profile for 25 randomized controlled trials included in this review.

Study	Quality assessment of evidence					
	**Limitation**	**Inconsistency**	**Indirectness**	**Imprecision**	**Risk of bias**	**Quality grading**
				detect significant difference)	blinding not mentioned)	
Kepka, et al.**(89)**	Serious limitation (sample size calculation was unclear)	No serious inconsistency	No serious indirectness	No serious imprecision	Very serious risk of bias (presence of selection bias; methods of blinding not mentioned)	⨁◯◯◯ Very low
Banerjee, et al.**(70)**	Serious limitation (sample size calculation was not mentioned)	No serious inconsistency	No serious indirectness	No serious imprecision	Very serious risk of bias (large losses to follow-up; lack of blinding)	⨁◯◯◯ Very low
Bronchetti, et al.**(68)**	Serious limitation (sample size calculation was not mentioned)	Serious inconsistency (the allocation of intervention and control was unclear)	No serious indirectness	No serious imprecision	Serious risk of bias (methods of blinding not mentioned)	⨁◯◯◯ Very low
Milkman, et al.**(59)**	Serious limitation (sample size calculation was not mentioned)	No serious inconsistency	No serious indirectness	No serious imprecision	None detected	⨁⨁⨁◯ Moderate
Staras, et al.**(48)**	No serious limitation	No serious inconsistency	No serious indirectness	No serious imprecision	Serious risk of bias (methods of blinding not mentioned)	⨁⨁⨁◯ Moderate
Milkman, et al.**(49)**	No serious limitation	No serious inconsistency	No serious indirectness	No serious imprecision	None detected	⨁⨁⨁⨁ High
Szilagyi, et al.**(61)**	No serious limitation	No serious inconsistency	No serious indirectness	No serious imprecision	None detected	⨁⨁⨁⨁ High
Okuno, et al.**(83)**	Serious limitation (sample size calculation was not mentioned)	No serious inconsistency	No serious indirectness	No serious imprecision	None detected	⨁⨁⨁◯ Moderate
Motta, et al.**(44)**	No serious limitation	No serious inconsistency	No serious indirectness	No serious imprecision	Serious risk of bias (methods of blinding not mentioned)	⨁⨁⨁◯ Moderate
Schmidtke, et al.**(52)**	No serious limitation	No serious inconsistency	No serious indirectness	No serious imprecision	None detected	⨁⨁⨁⨁ High
Panozzo, et al.**(65)**	No serious limitation	No serious inconsistency	No serious indirectness	No serious imprecision	Serious risk of bias (large losses to follow-up)	⨁⨁⨁◯ Moderate
Chen, et al.**(51)**	No serious limitation	No serious inconsistency	No serious indirectness	No serious imprecision	None detected	⨁⨁⨁⨁ High
Kuru, et al.**(88)**	No serious limitation	No serious inconsistency	No serious indirectness	No serious imprecision	Serious risk of bias (methods of blinding not mentioned)	⨁⨁⨁◯ Moderate
Freeman, et al.**(78)**	No serious limitation	No serious inconsistency	No serious indirectness	No serious imprecision	None detected	⨁⨁⨁⨁ High

Reñosa MDC, *et al. BMJ Global Health* 2021; 6:e006237. doi: 10.1136/bmjgh-2021-006237

**Supplementary Table 7. GRADE evidence profile for 25 randomized controlled trials included in this review.**						
**Study**	**Quality assessment of evidence**					
	**Limitation**	**Inconsistency**	**Indirectness**	**Imprecision**	**Risk of bias**	**Quality grading**
Cox, et al.**(90)**	No serious limitation	No serious inconsistency	No serious indirectness	No serious imprecision	None detected	⊕⊕⊕⊕ High
Frew, et al.**(81)**	No serious limitation	No serious inconsistency	No serious indirectness	No serious imprecision	Serious risk of bias (methods of blinding not mentioned)	⊕⊕⊕◯ Moderate
Maltz and Sarid**(66)**	No serious limitation	No serious inconsistency	No serious indirectness	No serious imprecision	Serious risk of bias (methods of blinding not mentioned)	⊕⊕⊕◯ Moderate

Reñosa MDC, *et al. BMJ Global Health* 2021; 6:e006237. doi: 10.1136/bmjgh-2021-006237
